# Guidance for Evidence-Informed Policies about Health Systems: Rationale for and Challenges of Guidance Development

**DOI:** 10.1371/journal.pmed.1001185

**Published:** 2012-03-06

**Authors:** Xavier Bosch-Capblanch, John N. Lavis, Simon Lewin, Rifat Atun, John-Arne Røttingen, Daniel Dröschel, Lise Beck, Edgardo Abalos, Fadi El-Jardali, Lucy Gilson, Sandy Oliver, Kaspar Wyss, Peter Tugwell, Regina Kulier, Tikki Pang, Andy Haines

**Affiliations:** 1 Swiss Tropical and Public Health Institute, Basel, Switzerland; 2 University of Basel, Basel, Switzerland; 3 McMaster Health Forum, Centre for Health Economics and Policy Analysis, Department of Clinical Epidemiology and Biostatistics, and Department of Political Science, McMaster University, Hamilton, Ontario, Canada; 4 Norwegian Knowledge Centre for the Health Services, Oslo, Norway, and Health Systems Research Unit, Medical Research Council of South Africa, Cape Town, South Africa; 5 Global Fund to Fight AIDS, Tuberculosis and Malaria, Geneva, Switzerland; 6 Harvard Kennedy School, Cambridge, Massachusetts, United States of America; 7 Centro Rosarino de Estudios Perinatales, Rosario, Argentina; 8 Department of Health Policy and Management, American University of Beirut, Beirut, Lebanon, and McMaster Health Forum, McMaster University, Hamilton, Ontario, Canada; 9 School of Public Health, University of Cape Town and Department of Global Health and Development, London School of Hygiene and Tropical Medicine, United Kingdom; 10 EPPI-Centre, Social Science Research Unit, Institute of Education, London, United Kingdom; 11 Centre for Global Health, Institute of Population Health, University of Ottawa, Ottawa, Ontario, Canada; 12 Innovation, Information, Evidence and Research, World Health Organization, Geneva, Switzerland; 13 Departments of Social and Environmental Health Research and of Nutrition and Public Health Research, London School of Hygiene & Tropical Medicine, London, United Kingdom

## Abstract

In the first paper in a three-part series on health systems guidance, Xavier Bosch-Capblanch and colleagues examine how guidance is currently formulated in low- and middle-income countries, and the challenges to developing such guidance.

**Summary Points**

- Weak health systems hinder the implementation of effective interventions; policies to strengthen such systems need to draw on the best available evidence.
- Health systems evidence is best delivered in the form of guidance embedded in policy formulation processes, but health systems guidance is poorly developed at present.
- The translation of research on problems, interventions, and implementation into decisions and policies that affect how systems are organised is one challenge facing the development of health systems guidance.
- The development of guidance that is timely and usable by the broad range of health systems stakeholders, and of methods to appraise the quality of health systems guidance, are additional challenges.
- Further research is needed to adapt existing approaches (e.g., those used in clinical guidelines) to produce meaningful advice that accounts for the complexity of health systems, political systems, and contexts.
- This is the first paper in a three-part series in *PLoS Medicine* on health systems guidance.

*This is one paper in a three-part series that sets out how evidence should be translated into guidance to inform policies on health systems and improve the delivery of clinical and public health interventions.*

## Introduction

Present trends suggest that many of the poorest countries in the world, including many in sub-Saharan Africa, will not meet the health-related Millennium Development Goals [1] (MDGs), especially MDG 4 (reducing under-five mortality) and MDG 5 (reducing maternal mortality) [2]. Even in those countries that are on track to meet health MDGs, striking inequities exist among countries and among socioeconomic groups within them [3], despite effective and cost-effective interventions being available to improve population health, including that of vulnerable groups [4]. Such interventions are delivered through health systems, which consist of “all organisations, people and actions whose primary intent is to promote, restore or maintain health” [5], but, in many settings, interactions between weakened health systems and the sometimes conflicting demands of single-disease intervention programmes are hindering the uptake and implementation of life-saving interventions [6]–[8]. A growing number of governments, international institutions, and funding agencies have therefore recognised the urgent need to coordinate and harmonise investments in health systems strengthening in low- and middle-income countries (LMICs) to provide universal social protection and effective coverage of essential health interventions [9].

Investments in health systems aim to “enhance [their] performance…for meeting the needs of patients and populations in an equitable and efficient manner” [10] while reducing the risk of impoverishment due to the costs of care [11]. However, although a number of broad principles have been proposed [12], there is no wide agreement on the operational definition of health systems strengthening [13], and it remains unclear how health systems can best be strengthened. Because the evidence base addressing this issue is patchy [14], health systems research has recently been identified as a priority [15] and its definition and scope have been outlined [16]. Indeed, the need for greater capacity to produce evidence to inform health systems strengthening was one of the drivers that led to the first global symposium on health systems research (Montreux, Switzerland, November 2010) [17], at which some of the issues developed in this article were presented and discussed.

Importantly, to be useful to policy makers, research evidence needs to be retrieved, its quality appraised, and the recommended options properly framed in the form of guidance. In an analogy with clinical practice guidelines (“systematically developed statements to assist practitioner and patient decisions about appropriate health care for specific clinical circumstances” [18]), we define health systems guidance as systematically developed statements produced at global or national levels to assist decisions about appropriate options for addressing a health systems challenge in a range of settings and to assist with the implementation of these options and their monitoring and evaluation (Box 1). We use the term “guidance” rather than “guidelines”, as health systems and the evidence on health systems are highly context sensitive. Health systems guidance statements refer to policy options that are accompanied by assessments of the quality of evidence supporting them and the potential for unintended harms, and by discussions of implementation and contextual issues.

**Box 1. Health Systems Guidance and Knowledge Translation**

Evidence-informed health systems guidance tackles health systems problems by:

- Framing health systems problems;
- Systematically retrieving, translating, and packaging the best available evidence on health systems interventions and implementation issues;
- Using this evidence to recommend and formulate—in a deliberative process—options to solve these problems and to inform policy-making, the level of decision-making where different courses of action are considered;
- Providing insights on the strategies that can be followed in order to implement and evaluate a given health systems policy.

Guidance needs to be transparent, systematic, and adapted to the local contexts. It therefore needs to use validated approaches, to consider all the available evidence and to assess its quality. It also needs to take into account local factors that may influence the effects of all options recommended and to address their feasibility.

Several global institutions have legitimate roles in producing guidance, often in response to requests from national decision makers (see the second paper in this series [23]). Thus, to avoid unnecessary duplication of the enormous efforts of guidance development, coordination is needed at the global level. However, as decisions on the options recommended are taken at national level, global guidance needs adaptation, ideally through national deliberative processes.

**Figure 1:**
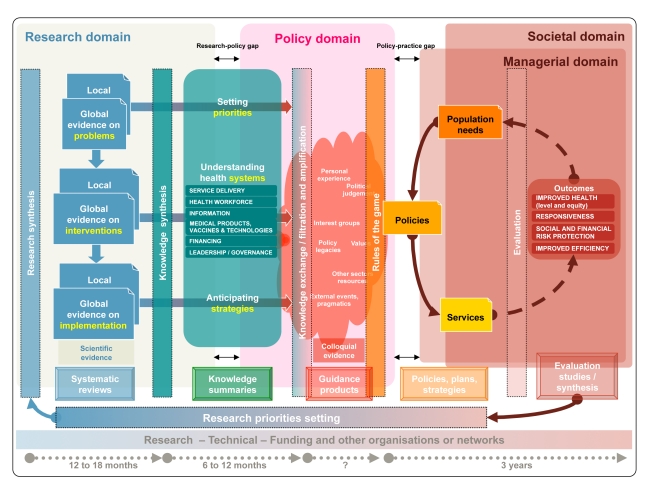
A generic knowledge translation framework across the research, policy, managerial, and societal domains. Vertical rectangles contain the methods or approaches to bridging each phase, the frames at the bottom indicate the products for each phase, and the concepts in between the vertical rectangles represent the different forms of knowledge. Systematic reviews are summarised into a unified body of knowledge that links priority problems with the effects of interventions and implementation strategies. Knowledge summaries support the deliberative process in which stakeholders develop guidance products that, in turn, result in policies for services and programmes arrangements. The outcomes of programmes and services are evaluated to ascertain the extent to which the needs of the population have been met. Evaluation should inform further research, in an iterative process.

The production of guidance is largely based on knowledge translation approaches that bridge the gap between research evidence and its application to policy-making [53],[54]. Figure 1 schematically represents these approaches across the research, policy, managerial, and societal domains.

The need for evidence-informed guidance on policies that impact health systems performance is widely accepted [19] and is one of the six priorities of the director-general of the World Health Organization (WHO) [5]. Moreover, a World Health Assembly resolution [20] recently urged member states to use evidence-based approaches to assess “country's health and health systems challenges” and to develop “evidence-based responses to evolving challenges and opportunities, and to involve all relevant stakeholders”. However, although well-established methods exist to develop clinical guidelines [21], there is little experience in developing health systems guidance and the process poses conceptual and methodological challenges related to the different types of evidence to be considered, the complexity of health systems and the pre-eminence of contextual issues. The current experience is mainly related to the development of different by-products of research syntheses and decision aids [22] targeted towards policy makers, rather than systematically and transparently developed guidance.

This paper, which is the first in a three-part series on health systems guidance [23],[24], aims to:

- Assess to what extent the need for health systems guidance is part of national policies and plans and how guidance is currently formulated by analyzing strategic health sector documents from LMICs;
- Describe the methodological challenges in outlining the approaches to produce health systems guidance and to suggest ways to address these challenges.

The second article in this series explores the challenge of linking guidance development and policy development at global and national levels and examines the range of factors that can influence policy development [23], and the third article explores the challenge of assessing how much confidence to place in evidence on health systems interventions [24].

## The Need for Health Systems Guidance in LMICs

To assess the need for guidance and how it is formulated, we scrutinised the use of guidance-related terms in national health policy and strategic documents from LMICs (Box 2). We found that the terms “guidance” or “guidelines” frequently appeared in strategic documents but were more often related to clinical matters than to health systems (Box 3).

**Box 2. Assessing Demand for Health Systems Guidance: Methods**

To assess the need of guidance and how it is formulated:

- Two authors examined all available documents written in English and French in the Country Planning Cycle Database [55], which has gathered documents that describe national health policies or strategies since 2005, for the following LMICs: Afghanistan, Bangladesh, Benin, Burkina Faso, Burundi, Cambodia, Central African Republic, Chad, Comoros, Congo DR, Eritrea, Ethiopia, The Gambia, Ghana, Guinea, Guinea Bissau, Haiti, Kenya, Korea DPR, Kyrgyzstan, Lao People's Democratic Republic, Liberia, Madagascar, Malawi, Mali, Mauritania, Myanmar, Nepal, Niger, Rwanda, Sierra Leone, Solomon Islands, Somalia, Tanzania, Togo, Uganda, Zambia, and Zimbabwe.
- The documents were searched using the terms “guideline(s)”, “guidance”, “guide(s)”, “recommendation(s)”, and their French equivalents and sentences or paragraphs where the terms appeared were extracted.
- One of the authors classified the statements extracted from the documents according to the area of interest (e.g., clinical, public health, health systems), health system component, the purpose of guidance (e.g., for setting standards, to guide policy decisions), the decisional scope of guidance (e.g., national, sub-national), guidance developers and sources (e.g., Ministries of Health, donor, United Nations agencies), guidance production, and the topics of guidance according to the WHO Health Systems Framework [5] (SI1).
- Of the 195 documents retrieved from the database, 157 dealing with specific programmes or strategies were excluded.
- Of the 661 statements retrieved in the 38 remaining documents, 161 were excluded because the term “guidance” was not used in the sense of decision-support (relevant for our analysis), but rather as generic advice or because the contexts where the terms appeared did not fit into any of the areas described above.

**Box 3. Assessing the Demand for Guidance: Findings**

The scope of guidance could be discerned in 63 statements from the included documents:

- 35 statements (56%) were national (i.e., supported decisions or activities implemented consistently across a country)
- 14 (22%) were sub-national
- 4 (6%) referred to international guidance
- 20 (32%) related to stakeholders, certain types of health facilities, or all levels of care.

The area of guidance could be identified in 407 statements:

- 201 statements (49.3%) referred to clinical issues
- 63 (15%) referred to public health issues
- 143 referred to miscellaneous areas (e.g., laboratory, management).

In 283 statements (either related to clinical, public health, or miscellaneous issues), references to one or more WHO health systems building blocks [5] included:

- 83 (29%) on governance and leadership (e.g., roles of governing bodies in producing or implementing guidance)
- 20 (7%) on financing (e.g., drug revolving funds)
- 53 (19%) on health workforce (e.g., training)
- 67 (24%) on medical supplies (including traditional medicines)
- 31 (11%) on information systems (e.g., data for measuring performance)
- 29 (10%) on service delivery (e.g., basic health care package)

Documents from eight countries had explicit statements suggesting guidance as a strategy to improve the “quality of care”. Guidance statements related to traditional medicine were found in The Gambia, Liberia, Rwanda, and Zimbabwe.

The main bodies developing guidance were international organisations (e.g., WHO), ministries of health, and special national committees usually linked to the ministries of health. Guidance production and use was mainly referred to in connection with high-level health sector entities, or at the sub-national level in decentralised structures, although similar terms were used in relation to implementation strategies or operational instructions. There were also statements emphasising the need for producing guidance, for making it accessible to users, and for reinforcing adherence. Finally, some documents explicitly related the development of guidance to the concepts of evidence-based health care.

Our search had several limitations. First, we may have missed some relevant documents. Second, statements do not necessarily reflect the status that governments or stakeholders ascribe to guidance. Finally, the terms used to refer to health systems guidance can differ between countries and across languages. Our findings nevertheless show that the term “guidance” appeared frequently in strategic documents. In its “technical” meaning (i.e., systematically developed statements to assist decisions), the term seems to be more often related to the clinical field rather than to health systems. This could be because the concept of formal guidance applied to health systems decisions is not yet well-established among policy makers or because there are only few examples of such guidance to draw from. However, the statements linking guidance in a more generic sense to health systems actions were plentiful in relation to the functions typically ascribed to ministries of health.

## Challenges in Outlining the Approaches to Produce Health Systems Guidance

To improve how WHO responds to requests for guidance on health systems and the quality of the guidance produced, WHO recently commissioned the production of a Handbook outlining approaches to develop health systems guidance [25]. A Task Force on Developing Health Systems Guidance (listed in the Acknowledgments section) was formed from WHO expert panels and from experts working in the field of health systems research at international and academic institutions and oversaw the drafting of the *Handbook for Developing Health Systems Guidance: Supporting Informed Judgements for Health Systems Policies* [25] (which was based on existing best practices for the development of clinical guidelines, on approaches and tools described in the literature, and on the expertise of the Task Force members) by reviewing and commenting on its content by email and through regular teleconferences and meetings in person. During the production of the Handbook, because evidence from research (for example, on the effectiveness and acceptability of health systems interventions) is not guidance and is insufficient for optimal decision-making [26], several conceptual and methodological challenges associated with the production of health systems guidance were identified. The writing group for this paper further considered these issues and produced this manuscript, which was finalised after several iterations of comments by the Task Force and external reviewers. Here, we discuss four specific challenges that were encountered during the production of the Handbook, namely:

- Research on effectiveness is typically articulated around health interventions, but policy decisions often relate to arrangements of the health system, services, and programmes and encompass multiple interventions packaged into a particular policy;
- Users and producers of guidance include a broad range of stakeholders who may not all be familiar with research methods;
- The production of guidance has to be timely in relation to the need and available capacity to implement it and consistent with countries' priorities;
- The quality of guidance needs to be appraised using transparent criteria; guidance then needs to be disseminated and promoted actively to facilitate its uptake.

### Translating Research on Problems, Interventions, and Implementation into Decisions on Policies and Services

Many processes, frequently involving iterations from research evidence into policy formulation and from policy evaluation into research prioritisation, are needed to bridge the gaps between research, policy, and practice. The complexity of these processes demands a dynamic framework [27] that is comprehensive and incorporates current thinking about evidence, policy formulation, and health systems such as the one shown in Figure 1.

Research is articulated around research questions that can be addressed using particular methods and is driven by research opportunities (e.g., funding), researchers' interests, and feasibility. By contrast, policy decisions and the managerial arrangements needed to put them into practice have to be responsive to population needs and integrated into complex health systems that go beyond the mere aggregation of single interventions. For example, the research question of whether lay health workers are effective in delivering specific health care interventions will become fully relevant for policy only when the essential components of the intervention (e.g., training of lay health workers), related actions (e.g., adaptations needed in the distributions of tasks of cadres), implementation issues (e.g., the preferences of potential clients), and the implications across other health system building blocks (e.g., adaptations to the health information sub-system that may be needed to capture the tasks undertaken by such workers) are all considered.

Ideally, implementation and contextual issues should be considered when conducting the systematic reviews of effectiveness that are needed to translate research into health systems guidance or in other types of reviews addressing them [28],[29]. Although there are frameworks to analyse barriers to implementation [30] and the applicability of evidence [31], many systematic reviews fail to consider issues pertaining to applicability and equity, or the complexity of interventions in relation to their technical feasibility [32]. This is partially due to the lack of this type of information in the primary research that underlies systematic reviews. Where this evidence is missing or scarce, efforts to search, appraise, and synthesise additional evidence on implementation issues will have to be made. It will also be important to consider how to address inequities in coverage of health care [30], which are pervasive in many countries.

The production of guidance not only entails putting research findings into context, but also entails taking a health systems perspective. New trends in health systems thinking advocate the construction of conceptual pathways that look at the interventions from a systems perspective, making explicit how interventions may trigger reactions in related components of the health system that may produce unintended consequences [33],[34]. For example, increasing the salary of health workers in HIV/AIDS treatment programmes may result in a reduction of the health workforce addressing other conditions who do not receive the same salary benefits.

Finally, it is important to note that the study designs for research (and the methods used to synthesise research findings) that explore different health systems components vary. For example, research exploring the governance and leadership components of health systems is likely to involve qualitative methods, whereas research on health care delivery could utilise both qualitative and quantitative methods (see also the third paper in this series [24]). Qualitative synthesis methods have addressed the nature of problems such as patient adherence to treatment for tuberculosis [35]. Syntheses of mixed method research can address policy-oriented questions using framework synthesis, a method that has been applied to qualitative and mixed methods research addressing research management [36], public health [37],[38], and workforce management [39].

### Producers and Users of Guidance

Although research evidence to inform guidance is generated through research synthesis (i.e., systematic reviews) and often uses complex statistical methods, guidance is typically produced through a deliberative process where evidence is interpreted and contextualised (Figure 1, Policy domain). During the deliberative process, which can include a wide range of stakeholders, potential users, and beneficiaries of guidance, knowledge is exchanged, filtered (stakeholders decide on the relevance of evidence), and amplified (stakeholders stress evidence consistent with their views). Colloquial (non-research) evidence (e.g., tacit or experiential knowledge) helps to interpret or contextualise research evidence and addresses issues for which research evidence is not available but that may play a role in the decisions (e.g., considerations about the political implications of decisions, or about potential vested interests of stakeholders) [40]. Making colloquial evidence explicit adds transparency to the guidance development process.

Typically, users and producers of guidance, who may include economists, managers, administrators, social scientists and other professional groups as well as elected politicians, come from very different research traditions or none at all and have very different approaches to research and decision-making [41]. Therefore, it is probably unrealistic to expect that they will all be familiar with issues such as assessing the quality or appropriateness of systematic reviews [42]. Furthermore, even in the ideal situation where policy makers are familiar with research methods, they may choose to ignore evidence that creates uncertainty, questions conventional knowledge, ignores local context [43], or that is not consistent with pre-determined ideas of preferred policy options.

Although evidence on the effects of health systems interventions is relatively scarce, there are renewed efforts to increase availability and accessibility of this evidence [22],[44],[45]. Such evidence should be presented in user-friendly formats (e.g., minimising technicalities such as complex statistical outputs) to increase its accessibility when producing guidance and to facilitate appropriate interpretation by those without a strong research background. Finally, the preferences of different decision makers for how evidence and guidance should be presented should be taken into account.

### Timeliness in the Production of Guidance

Timeliness in the production of evidence and guidance is critical, but systematic reviews can take between one and two years to complete depending on the availability of new evidence, and guidance development can take a year or more [46]. The bottom of Figure 1 also shows the potentially long time frames for the policy decision processes and the evaluation of policies. Furthermore, guidance needs to be regularly updated in the light of new evidence or changes and modifications to existing, previously accepted evidence.

Without timely production of research synthesis and guidance, the gap between research, policy, and practice cannot be bridged, even if primary research is available. Alternative approaches and methods are therefore needed to reduce the lengthy time frames needed for producing systematic reviews and for translating the findings of reviews into guidance. Approaches that may be useful include rapid assessment methods [47], the use of text mining to speed the search for publications [48], and the adaptation of existing guidance that can be institutionally endorsed by the organisations producing or using it [47].

### Appraisal, Dissemination, and Implementation of Guidance

The quality of guidance has to be assessed to ascertain the extent to which the guidance used state-of-the-art and validated methods during its development and is, therefore, balanced and reliable in relation to the evidence that informs it. The AGREE instrument (Appraisal of Guidelines Research and Evaluation) and its recent revision (AGREE II) is used to assess the quality of clinical guidelines but could be adapted to assess health systems guidance. The AGREE instrument, which consists of 23 items, aims to assess the quality of guidelines, to provide a methodological strategy for the development of guidelines, and to inform guideline developers on what and how information ought to be reported in guidelines [49]. Many of its items are applicable to both clinical guidelines and health systems guidance, but some need minor adaptation for the latter. For example, item 16 in the AGREE II instrument mentions the “different options for the management of the condition or health issue are clearly presented” whereas in health systems guidance this would need to be reworded to indicate “different options for addressing the health system topic are clearly presented”. In addition, there should be specific reference to the need for health systems guidance to refer to the contextual factors that would determine the extent to which research evidence is applicable in specific circumstances.

Passive dissemination of guidance will not ensure its uptake by potential users [50], particularly if it lacks relevance to local or national situations. There are several proven approaches for the effective dissemination of research evidence and the promotion of clinical guidelines (e.g., distribution of educational materials, educational meetings and outreach visits, involvement of local opinion leaders, audit and feedback) that have moderate effects on outcomes [51]. Some of these approaches may also be relevant to health systems guidance, although the challenges relating to dissemination and uptake for these two types of guidance are different. At a national level, a relatively small number of decision makers may need to be influenced to integrate guidance into national health policies and plans. Alternatively, sub-national and local decision makers may be in a better position to influence guidance implementation, particularly in more decentralised systems. “Policy briefs” and “policy dialogues” are examples of tools used to improve the contextualisation and utilisation of guidance [41] (discussed further in the second article in this series [23]). Although uptake of guidance depends on how it is presented and disseminated, additional factors may increase the utilisation of scientific evidence by policy-makers. These include early, informal interactions between researchers and policy makers, the relevance and timeliness of evidence, and the consistency between the evidence and the recommended options on the one hand, and the beliefs, values, interests, or political goals of policy makers on the other [52].

## Conclusions

We found that high-level policy and strategic health sector documents frequently mention the need for the development of health systems guidance but suggest that there may still be limited awareness that health systems guidance can provide key inputs into policy-making and that the development of such guidance requires systematic and transparent approaches, inspired by the development of clinical guidelines. However, given the growing amount of evidence on health systems and the current initiatives to address methodological challenges to produce health systems guidance (e.g., the *Handbook for Developing Health Systems Guidance* [25]), the international health community at all levels is likely to be faced with increasing demands for guidance on health systems issues.

The approaches we identified for producing guidance for health systems typically mirror validated methods developed for clinical guidelines but with some important differences. Specifically, to develop health systems guidance that is relevant and useful to decision makers, it is necessary to acknowledge and to find ways of incorporating the complex interrelations of the system components, and the numerous contextual factors that may influence the effectiveness of interventions, particularly their effects on disadvantaged populations. These issues are discussed further in the second paper in this series [23]. The uptake of guidance by decision makers and the additional complexity of the decision-making process itself are also important.

Health systems guidance has the potential to improve decision-making and enable more efficient use of resources with consequent improvements in the health of populations. However, such guidance needs rigorous and transparent processes of production and evidence-based approaches to ensure its dissemination and uptake. These processes and approaches are still at a rudimentary stage of development. Importantly, the availability of health systems guidance should encourage better and more comprehensive health systems research. This, in turn, should foster more cross-disciplinary approaches to studying the dynamic interactions within complex health systems and thus help to develop new methods to effectively translate health systems evidence into usable policy guidance that is relevant to stakeholders at global and national levels, that takes into account social and ethical imperatives, and that recognises the complexity of health systems and political systems within which such guidance is introduced.

## Supporting Information

Alternative Language Summary Points S1 **Translation of the Summary Points into Spanish by Xavier Bosch-Capblanch**
(DOC)

Alternative Language Summary Points S2 **Translation of the Summary Points into French Bruno Clary, William Lenoir, and Lise Beck**
(DOC)

Alternative Language Summary Points S3 **Translation of the Summary Points into Portuguese by Bruno Viana**
(DOC)

Alternative Language Summary Points S4 **Translation of the Summary Points into Arabic by Fadi El-Jardali**
(DOC)

Table S1 **Characteristics of guidance in health policy or strategy documents analysed with examples of quotations**
(DOC)

## Abbreviations

AGREE: Appraisal of Guidelines Research and Evaluation
LMIC: low- and middle-income country
MDG: Millennium Development Goal
WHO: World Health Organization
