# Supplementary material for: Guidance for Evidence-Informed Policies about Health Systems: Rationale for and Challenges of Guidance Development
Source: PLoS Med. 2012 Mar 6;9(3):e1001185. doi: 10.1371/journal.pmed.1001185 (PMC3295823; doi:10.1371/journal.pmed.1001185)
Supplement: Alternative Language Summary Points S1 — Translation of the Summary Points into Spanish by Xavier Bosch-Capblanch (DOC) [file pmed.1001185.s001.doc]

PLOS 1

Resumen

- Los sistemas de salud débiles hacen peligrar la implementación de intervenciones efectivas; las políticas para reforzar estos sistemas deben basarse en la mejor evidencia disponible.
- La mejor manera de presentar evidencias sobre sistemas es a través de guías incorporadas en los procesos de formulación de políticas; sin embargo, actualmente casi no se han desarrollado guías sobre sistemas de salud.
- La traducción de las investigaciones acerca de los problemas, de las intervenciones y de la implementación de las mismas en políticas que afecten la organización de los sistemas es un reto afectando el desarrollo de guías sobre sistemas de salud.
- Otros retos son el desarrollo de guías producidas oportunamente y que sean utilizables por el amplio abanico de partes interesadas, así como los métodos para evaluar la calidad de las guías sobre sistemas.
- Es preciso llevar acabo investigaciones adicionales para adaptar los enfoques existentes (por ejemplo, los utilizados en guías clínicas) para producir recomendaciones significativas que tengan en cuenta la complejidad de los sistemas de salud, de los sistemas políticos y de sus contextos.

PLOS 2

Resumen

- Los factores contextuales son extremadamente importantes para formar las decisiones sobre los sistemas de salud, y los políticos tienen que clarificar los pros y contras de diferentes opciones antes de adoptar guías específicas sobre sistemas de salud.
- Es necesaria la división del trabajo entre desarrolladores de guías globales, formuladores de políticas globales, productores de guías nacionales y formuladores de políticas nacionales para promover políticas sobre sistemas de salud informadas por evidencias.
- El valor añadido de un panel de expertos a nivel global encargado de la producción de guías sobre sistemas de salud sería asegurar que el resultado de su trabajo se puede usar en el desarrollo de políticas a los niveles global y nacional, y para la producción de guías a nivel nacional.
- A los niveles global y nacional se precisan análisis rigurosos de los sistemas de salud y de los sistemas políticos para apoyar el desarrollo de guías y de políticas.
- Es preciso llevar a cabo investigaciones adicionales en relación con la división del trabajo en la producción de guías y en el desarrollo de políticas y sobre los marcos conceptuales donde situar los análisis de sistemas y políticos.

PLOS 3

Resumen

- La valoración del nivel de confianza que se puede depositar en diferentes tipos de evidencias resultantes de la investigación es fundamental para informar los juicios relativos a las opciones políticas para abordar los problemas de los sistemas de salud.
- Es particularmente importante que estas valoraciones tengan abordajes sistemáticos y transparentes dada la complejidad de muchas intervenciones sobre sistemas de salud.
- Existen herramientas para valorar el nivel de confianza que se puede depositar en diferentes tipos de evidencias resultantes de la investigación necesarias para sostener las fases del proceso de elaboración de políticas; estando más desarrolladas las herramientas que valoran las evidencias en relación a los efectos de las intervenciones.
- Aun es preciso desarrollar herramientas para asistir los juicios relativos a las evidencias de las revisiones sistemáticas acerca de otros factores clave, tales como la aceptabilidad de las opciones políticas por las partes interesadas, la viabilidad de la implementación y la equidad.
- También es preciso investigar las maneras de desarrollar, estructurar y presentar las opciones políticas en las guías globales de sistemas de salud.

Xavier Bosch-Capblanch, Swiss TPH
