# Supplementary material for: Guidance for Evidence-Informed Policies about Health Systems: Rationale for and Challenges of Guidance Development
Source: PLoS Med. 2012 Mar 6;9(3):e1001185. doi: 10.1371/journal.pmed.1001185 (PMC3295823; doi:10.1371/journal.pmed.1001185)
Supplement: Alternative Language Summary Points S2 — Translation of the Summary Points into French Bruno Clary, William Lenoir, and Lise Beck (DOC) [file pmed.1001185.s002.doc]

Résumé 1

- Les systèmes de santé faibles entravent la mise en œuvre des interventions efficaces; les politiques visant à renforcer de tels systèmes doivent se baser sur les meilleures preuves factuelles disponibles.
- La meilleure façon de fournir des preuves factuelles sur les systèmes de santé se fait au travers de guides intégrés dans le processus d’élaboration des politiques. Toutefois, à l’heure actuelle, les guides sur les systèmes de santé demeurent peu développés.
- Le passage de la recherche, portant sur les problèmes, les interventions et la mise en œuvre, aux décisions et aux politiques qui affectent la façon dont les systèmes sont organisés, est un défi auquel le développement des guides sur les systèmes de santé doit faire face.
- L’élaboration de guide, opportuns et utilisables par une majeure partie des acteurs des systèmes de santé, et de méthodes pour évaluer la qualité des guides portant sur les systèmes de santé, est un défi supplémentaire.
- Des recherches complémentaires sont nécessaires pour adapter les approches existantes (par exemple celles utilisées dans les guides cliniques) afin de produire des recommandations avisés qui tiennent compte de la complexité des systèmes de santé, des systèmes politiques et du contexte.

Résumé 2

- Les facteurs contextuels sont extrêmement importants dans la formulation des décisions portant sur le système de santé et les décideurs politiques doivent peser le « pour » et le « contre » des différentes options avant d’adopter des directives spécifiques sur les systèmes de santé
- Une claire répartition des taches entre ceux qui développent des directives aux niveaux global ou national, entre ceux qui développent des politiques aux niveaux global ou national, est nécessaire pour soutenir les décisions politiques éclairées sur les systèmes de santé, informées par des preuves factuelles, .
- La meilleure valeur ajoutée d’une commission chargée de développer des guides sur les systèmes de santé au niveau global consisterait à s’assurer que les résultats de son travail puisse être utilisés dans la définition des politiques au niveau national et global, et dans l’élaboration de guides au niveau national.
- Des analyses rigoureuses des systèmes de santé et des politiques sont nécessaires au niveau global et national afin de soutenir l’élaboration des guides et des politiques.
- De plus amples recherches sont nécessaires sur la répartition du travail dans le développement de guides et de politiques ainsi que sur les cadres conceptuels appuyant les analyses des systèmes et des politiques.

Résumé 3

- Evaluer dans quelle mesure il faut faire confiance aux différents types de preuves factuelles est primordial pour rendre un jugement avisé sur les choix politiques visant à solutionner les problèmes des systèmes de santé.
- Des approches systématiques et transparentes lors de telles évaluations sont particulièrement importantes, compte tenu de la complexité des interventions portant sur les systèmes de santé.
- Des instruments utiles sont disponibles pour évaluer le degré de confiance à placer dans les différents types de preuves factuelles nécessaires pour soutenir les différentes étapes du processus décisionnel lors de l’élaboration des politiques ; des instruments pour l’évaluation des preuves factuelles portant sur l’efficacité sont davantage développés.
- Des instruments doivent être développés pour assister les jugements, en tenant compte des preuves factuelles provenant d’études systématiques sur d’autres facteurs clés comme l’acceptation des différentes options politiques par les différents acteurs, la faisabilité de la mise en place des interventions et l’équité.
- Des recherches sont également nécessaires sur la manière d’élaborer, structurer et présenter les différentes options des politiques dans les guides globales sur les systèmes de santé.

Bruno Clary and William Lenoir, Swiss TPH
