# Supplementary material for: Guidance for Evidence-Informed Policies about Health Systems: Rationale for and Challenges of Guidance Development
Source: PLoS Med. 2012 Mar 6;9(3):e1001185. doi: 10.1371/journal.pmed.1001185 (PMC3295823; doi:10.1371/journal.pmed.1001185)
Supplement: Alternative Language Summary Points S3 — Translation of the Summary Points into Portuguese by Bruno Viana (DOC) [file pmed.1001185.s003.doc]

PLOS 1

Sumário

Os sistemas de saúde frágeis atrasam a implementação de intervenções eficazes; as políticas que visam reforçar estes sistemas necessitam de recorrer às melhores evidências disponíveis.

As evidências acerca dos sistemas de cuidados de saúde são melhor utilizadas sob formas de guias incorporadas no processo de elaboração de políticas, embora, actualmente, as guias sobre sistemas de saúde estejam pouco desenvolvidas.

Um dos desafios para o desenvolvimento de guias sobre os sistemas de saúde é a tradução da pesquisa sobre os problemas, intervenções e implementação para decisões e políticas que influenciam como os sistemas são organizados.

O desenvolvimento de guias oportunas e utilizáveis pelos diversos intervenientes dos sistemas de saúde, bem como de métodos de avaliação das guias, é um desafio adicional.

É necessária mais pesquisa para adaptar os métodos já existentes (por exemplo, aqueles utilizados nas guias clínicas) no sentido de produzir recomendações válidas que tenham em conta a complexidade dos sistemas de saúde, sistemas políticos e conjunturas.

PLOS2

Sumário

Os factores conjunturais são extremamente importantes na toma de decisões acerca dos sistemas de saúde, e os políticos necessitam de avaliar todos os prós e contras das diferentes opções antes de adoptar guias específicas para os sistemas de saúde.

A divisão de trabalho entre os responsáveis por desenvolver as orientações globais, políticas globais, orientações nacionais e políticas nacionais é necessária para sustentar as políticas informadas pela evidência sobre os sistemas de saúde.

Um painel de profissionais responsável pela elaboração de orientações globais para os sistemas de saúde poderia melhor acrescentar o seu valor ao assegurar que os resultados possam ser utilizadas para o desenvolvimento de políticas globais e nacionais, bem como para o desenvolvimento de guias nacionais.

São necessárias análises rigorosas aos sistemas de saúde e aos sistemas políticos, a nível nacional e internacional, de maneira a apoiar o desenvolvimento de guias e políticas.

É necessária pesquisa adicional acerca da divisão do trabalho em desenvolvimento de guias e desenvolvimento de políticas e acerca dos enquadramentos que sustentam as análises aos sistemas de saúde e políticos.

PLOS3

Sumário

É fundamental avaliar o grau de confiança a colocar nos diferentes tipos de evidências obtidas na pesquisa de forma a fundamentar decisões relativas a opções políticas que respondem aos problemas existentes nos sistemas de saúde.

Dado a complexidade de muitas intervenções nos sistemas de saúde, é de particular importância que estas avaliações tenham uma abordagem sistemática e transparente.

Estão disponíveis ferramentas úteis para avaliar o grau de confiança a colocar nos diferentes tipos de evidências obtidas na pesquisa que servirão de suporte a diferentes etapas do processo de elaboração de políticas; as mais desenvolvidas são aquelas que avaliam a evidência de eficácia das intervenções.

É necessário desenvolver ferramentas para assessorar decisões relativamente a evidências provenientes de revisões sistemáticas de outros fatores, como a aceitabilidade das opções políticas pelos intervenientes, viabilidade da implementação e equidade.

É também necessária pesquisa sobre modos de desenvolver, estruturar e apresentar opções políticas dentro das guias globais dos sistemas de saúde.

Bruno Viana, Swiss TPH
