# Supplementary material for: Guidance for Evidence-Informed Policies about Health Systems: Rationale for and Challenges of Guidance Development
Source: PLoS Med. 2012 Mar 6;9(3):e1001185. doi: 10.1371/journal.pmed.1001185 (PMC3295823; doi:10.1371/journal.pmed.1001185)
Supplement: Alternative Language Summary Points S4 — Translation of the Summary Points into Arabic by Fadi El-Jardali (DOC) [file pmed.1001185.s004.doc]

Guidance for Evidence-Informed Policies about Health Systems: Rationale for and Challenges of Guidance Development

**نقاط موجزة**

- إن الأنظمة الصحية الضعيفة تعيق تنفيذ التدخلات الفعّالة؛ وإن السياسات الهادفة لتدعيم مثل هذه الأنظمة يجب أن تستمدّ من أفضل الأدلة والبيّنات المتوفّرة.

- لعلّ من أفضل طرق تقديم بيّنات الأنظمة الصحّية هو تضمينها في آليات صنع السياسات؛ ولكن في الوقت الحاضر، فإن عملية إرشاد الأنظمة الصحية ضعيفة وتفتقر إلى آلية تطوير.

- تواجه عملية إرشاد الأنظمة الصحية تحدياً في كيفية ترجمة الأبحاث المتعلقة بالمشاكل والتدخلات وآليات التطبيق إلى قرارات وسياسات تؤثر على بنية الأنظمة الصحية وسير العمل فيها.

- ومن التحديات الأخرى أيضاً في هذا المجال، نذكر القدرة على تقديم الإرشادات والمشورة في الوقت المناسب وبصيغة قابلة للاستخدام من قبل أصحاب الشأن والمعنيين بالأنظمة الصحية على اختلاف خلفياتهم ومناصبهم؛ وتحدي القدرة على تطوير آليات تقييم جودة المشورة والإرشادات المقدمة في الأنظمة الصحية.

- هناك حاجة إلى مزيد من الأبحاث حول تكييف المقاربات الموجودة (مثل تلك المستخدمة في الإرشادات السريرية والعيادية) لكي تنتج مشورة ذات معنى في الإطار الأكثر تعقيداً للأنظمة الصحية والأنظمة السياسية وبيئات العمل فيها.
