# Supplementary material for: Guidance for Evidence-Informed Policies about Health Systems: Rationale for and Challenges of Guidance Development
Source: PLoS Med. 2012 Mar 6;9(3):e1001185. doi: 10.1371/journal.pmed.1001185 (PMC3295823; doi:10.1371/journal.pmed.1001185)
Supplement: Table S1 — Characteristics of guidance in health policy or strategy documents analysed with examples of quotations (DOC) [file pmed.1001185.s005.doc]

Table S1. Characteristics of guidance in health policy or strategy documents analysed with examples of quotations.

| **Characteristics** | **Quotation** | **Source** |
| --- | --- | --- |
| **Concept or purpose of guidance** | | |
| To define problems | “L’analyse de la situation est faite selon une grille d’analyse adaptée du «methodological guidelines for sectoral analysis in health»” | Haiti 2005 |
| To guide policies | “A decentralized and deconcentrated service delivery will be supported through strong government guidance” | Cambodia 2008 |
|  | “The national Ghana Health System, advised by district and regional authorities, agrees operational priorities at the district level, reflecting local health needs, within national strategic guidelines” | Ghana 2007 |
| To ensure compliance with regulations | “Le guide de contractualisation avec les ONG” (The guide for contracting NGOs [author’s translation]) | Mauritania 2005 |
|  | “develop regulatory guidelines and standard operating procedures” | Sierra Leone 2009 |
| To set standards | “commodities […] are sufficiently available as per the standards laid down in medical procedures and guidelines” | Kenya 2005 |
|  | “percentage of health facilities with stock-outs of essential drugs as defined by Ministry of Health guidelines” | Rwanda 2009 |
| To guide implementation operations | “set forth clear guidance for program implementation” | Afghanistan 2008 |
|  | “As stated in the implementation guidelines” | Ethiopia 2008 |
| To support managerial tasks | “Comprehensive human resource management guidelines elaborated and adopted” | Kenya 2005 |
|  | “annual planning and budgeting guidelines issued by the Ministry of Health” | Zambia 2005 |
| **Scope** | | |
| International organisations | “To update protocols for addressing each component of maternal nutrition as international guidelines” | Korea PDR 2006 |
|  | “The Ministry will enforce the standardization of basic equipment by adapting WHO Equipment Guidelines to the Liberian context” | Liberia 2006 |
| National | “All national health programmes address quality through their treatment guidelines and protocols” | Ethiopia 2008 |
|  | “The main broad strategies for community health care project are formulated through PHC approach reflecting the national health policy guidelines and national population policy guidelines” | Myanmar 2006 |
| Sub-national | “Development and use of district planning guidelines” | Sierra Leone 2009 |
| Health facilities | “development of management guidelines for hospitals and training institutions” | Zambia 2005 |
| Other | “Establish the health policy framework: This includes: issuing annual strategic direction and planning guidelines for itself and for other stakeholders” | Kenya 2005 |
|  | “planning and implementation guidelines for each programme” | Zambia 2005 |
| **Guidance developers and sources** | | |
| Ministries at national level | “The Ministry of Public Health […] is committed to set policies, standards and guidelines” | Afghanistan 2008 |
|  | “the Ministry of Health and Social Welfare will develop new policies, legislation and operational guidelines” | Tanzania 2009 |
| Committees | “The purpose of the national Reproductive Health (RH)steering committee is to provide policy guidance for all RH activities in Eritrea” | Eritrea 2006 |
|  | “For purposes of policy guidance a multidisciplinary National Health Advisory Council (NHAC) shall be established” | Liberia 2006 |
|  | “The Health Policy Advisory Committee (HPAC) has proved beneficial in providing overall policy guidance to the sector” | Uganda 2005 |
| Sub-national | “Most regions have now passed health proclamations and have issued directives and guidelines” | Ethiopia 2008 |
| International organisations | “The infection control practices in health care facilities will be developed according to the WHO'S practical guidelines for infection control” | Myanmar 2006 |
|  | “To review WHO/ILO/UNESCO guidelines on Community Based Rehabilitation for Zimbabwe” | Zimbabwe 2008 |
| Other | “A key challenge is the long and complicated international competitive tendering procedures of the World Bank procurement guidelines” | Ethiopia 2008 |
|  | “Recruit technical assistance to review and develop regulatory guidelines and standard operating procedures” | Sierra Leone 2009 |
| **Guidance production and use** | | |
| Development | “Elaborer un guide de bonnes pratiques de maintenance et de gestion durable des infrastructures” (To develop a best practice guideline for maintenance and management of infrastructures [author’s translation]) | Mali 2008 |
|  | “Developing guidelines for revolving drug funds by doing advocacy meeting to administration authorities and heads of departments of health from central level” | Myanmar 2006 |
| Availability | Regular up-to-date quality assurance policies, strategies, protocols and guidelines are still lacking or not properly communicated throughout the sector. | Rwanda 2009 |
|  | “Lack of standards and guidelines for data collection, analysis and reporting” | Sierra Leone 2009 |
|  | “Guidelines and diagnostic capacity, drugs and supplies must be available at health facilities” | Tanzania 2009 |
|  | “Make health promotion guidelines available to other stakeholders” | Zimbabwe 2008 |
| Dissemination and use | “Develop an official dissemination process for all documentation and guidelines” | Ethiopia 2008 |
|  | “Although standard guidelines and norms have been put in place, it remains a major challenge to implement them due to the human resource gaps at all levels” | Ethiopia 2008 |
|  | “Efforts need to be enhanced in the dissemination and enforcement of compliance with recognized/recommended treatment guidelines and prescriptions” | Zambia 2005 |
| **Topics: Health systems framework (building blocks)** | | |
| Governance and leadership | “Develop a cohesive Public-Private Partnership policy and guidelines for sustainable health care” | Sierra Leone 2009 |
|  | “Implementing the guidelines for the Private Not-For-Profit and Private Health Practitioners sub-sector to promote the partnership at Local Government level” | Uganda 2005 |
| Financing | A system of collection, retention and utilisation of “user fees” at all public health facilities […] will be established and for this a set of guidelines developed. | Bangladesh 2008 |
|  | “annual planning and budgeting guidelines issued by the MOH” | Zambia 2005 |
| Health workforce | “Provide guidelines for training of Medical Officers” | The Gambia 2007 |
|  | “The Ministry will produce guidelines for the hiring of expatriate health professionals” | Liberia 2006 |
|  | “Policy and guidelines we have developed for human resources development and management guidance on training and deployment, other policy documents were also developed in the area of reproductive health, TB Malaria and diagnostics” | Malawi 2006 |
| Medical products, vaccines and technologies | “To review existing policies and develop new policies and guidelines with respect to medicines, medical supplies and equipment, vaccines, health technologies and logistics” | Sierra Leone 2009 |
|  | “Guidelines and standard operating procedures for infrastructure maintenance” | Tanzania 2009 |
| Information | “Incorporate the Essential Health Service Package into planning, budgeting and M&E guidelines” | Ethiopia 2008 |
|  | “La préparation et la distribution de guides tant pour la surveillance  épidémiologique que pour les interventions en cas d’épidémie” (the preparation and distribution of guidelines for both epidemiological surveillance and in case of epidemic (author’s translation]) | Guinea 2002 |
|  | “Health information is important for monitoring the performance of the health sector. During HSSP I, guidelines and generic data analysis formats for all levels were developed and distributed in order to improve the analysis and interpretation of HMIS data” | Uganda 2005 |
| Service delivery | “The policies and guidelines in these documents are reflected in the Minimum Package of Activities” | Cambodia 2008 |
|  | Periodic update of tools and guidelines for improving access to medicines | Ghana 2007 |
